# Primary kidney malignant epithelioid angiomyolipoma: Two cases report and review of literature

**DOI:** 10.1097/MD.0000000000011805

**Published:** 2018-08-10

**Authors:** Rui Zhan, Yan-Qing Li, Chun-Yan Chen, Han-Yu Hu, Chun Zhang

**Affiliations:** a Department of Pathology, First People's Hospital of Wujiang District, Suzhou; b Department of Pathology, Chongqing Corps Hospital of Chinese People's Armed Polices, Chongqing, P.R. China.

**Keywords:** angiomyolipoma, EAML, epithelioid, kidney, malignant

## Abstract

**Rationale:**

Epithelioid angiomyolipoma (EAML) is a subtype of angiomyolipoma with malignant potential. A diagnosis of malignant EAML of the kidney is based on extrarenal metastasis, and predicting early transformation is difficult. To propose criteria for indicators of malignant transformation, herein we report 2 cases and review 17 cases reported in the literature (2000–2017).

**Patient concerns:**

Tumor of the kidney was determined in 2 patients, and tissues after nephrectomy were pathologically, histologically, and immunochemically examined.

**Diagnosis:**

Malignant EAML.

**Intervention:**

The 2 present patients were treated with nephrectomy only.

**Outcomes:**

Case 1 involved a 48-year-old woman with a 7.5-cm solid mass in the right kidney who underwent nephrectomy. CT detected a mass in the liver after 13 months, which was speculated to be metastasis from the kidney lesion. Case 2 involved a 62-year-old man with a 7-cm cystic solid mass in the left kidney who accepted nephrectomy and at 10 months post-surgery lived with no disease. Both cases presented a large tumor, atypical epithelioid cells, mitotic figures, and necrosis; tested positive for melanocytic markers (HMB45, MelanA).

**Lessons:**

The literature review of malignant EAML led to the identification of 8 malignant features: size ≥5 cm; metastasis; infiltration; necrosis; ≥50% atypical epithelioid cells; cytologic atypia; atypical mitosis; and vessel invasion. The co-existence of at least 5 of these is proposed to indicate malignant EAML. Features of our 2 new cases of primary malignant EAML of the kidney matched these criteria. Our proposal of criteria for predicting malignant feature, based on 2 new cases and 17 cases in the literature, should aid understanding and avoid misdiagnosis. Nephrectomy is currently the common treatment strategy for malignant EAML, but more effective treatment strategies are needed to provide a better prognosis for patients.

## 1 Introduction

Perivascular epithelioid cell neoplasm (PEComa) was first described in 1943, and in 1992 was proposed as a particular form of perivascular epithelioid cell tumor, with a positive marker being the monoclonal antibody HMB (human melanoma black)-45.^[1]^ PEComas originate from mesenchymal tissue and are characterized by perivascular epithelioid cells with melanocytic and myoid differentiation. PEComas types comprise the following: angiomyolipoma (AML), clear cell sugar tumor of the lung, lymphangioleiomyomatosis, clear cell myomelanocytic tumor, and some tumors that arise from unusual sites but which are not otherwise specified.^[2]^ The World Health Organization in 2002 classified PEComas as originating either from soft tissue or bone, based on the location of the tissue.^[3]^

Kidney AML is a common benign PEComa that consists of blood vessels, smooth muscle, and matured adipose tissue. Epithelioid AML (EAML) of the kidney is an unusual subtype of AML that is potentially malignant. EAML is mainly composed of epithelioid cells with abundant eosinophilic or granular cytoplasm, round to oval nuclei, and prominent nucleoli.^[4]^ Some studies have suggested that malignant progression of EAML may be predicted by the percentage of epithelioid cells, and <10, 80 to 95, and 95% epithelioid cells were associated with no, low (5%), and high progression rates (51.5%), respectively.^[5–7]^ However, it is difficult to make a definitive diagnosis of primary kidney malignant EAML, because there are no standardized judgement criteria based on clinicopathology.

To propose definitive judgement criteria for indicators of malignant EAML of the kidney, we report herein the detailed clinicopathological, morphological, and immunohistochemical features of 2 cases that were treated in our clinic. Moreover, the clinicopathological features, morphological features, therapy strategies, and prognoses of 17 cases from previous reports are discussed. From these, we determined 8 features of malignant EAML of the kidney, and propose that the coexistence of ≥5 of these 8 features indicate malignant EAML.

### 1.1 Case reports

#### 1.1.1 Case 1

A 48-year-old woman was found to have a solid mass in the right kidney, via ultrasonography during a regular physical examination. She did not complain about backache, abdominal pain, urinary irritation, hematuria, or dysuria. She had no history of tuberous sclerosis (TSC). The physical examination showed no eminence or tenderness in the costovertebral angle, hypochondriac point, or ureteral point. Laboratory examination did not show any abnormality.

**Figure 1:**
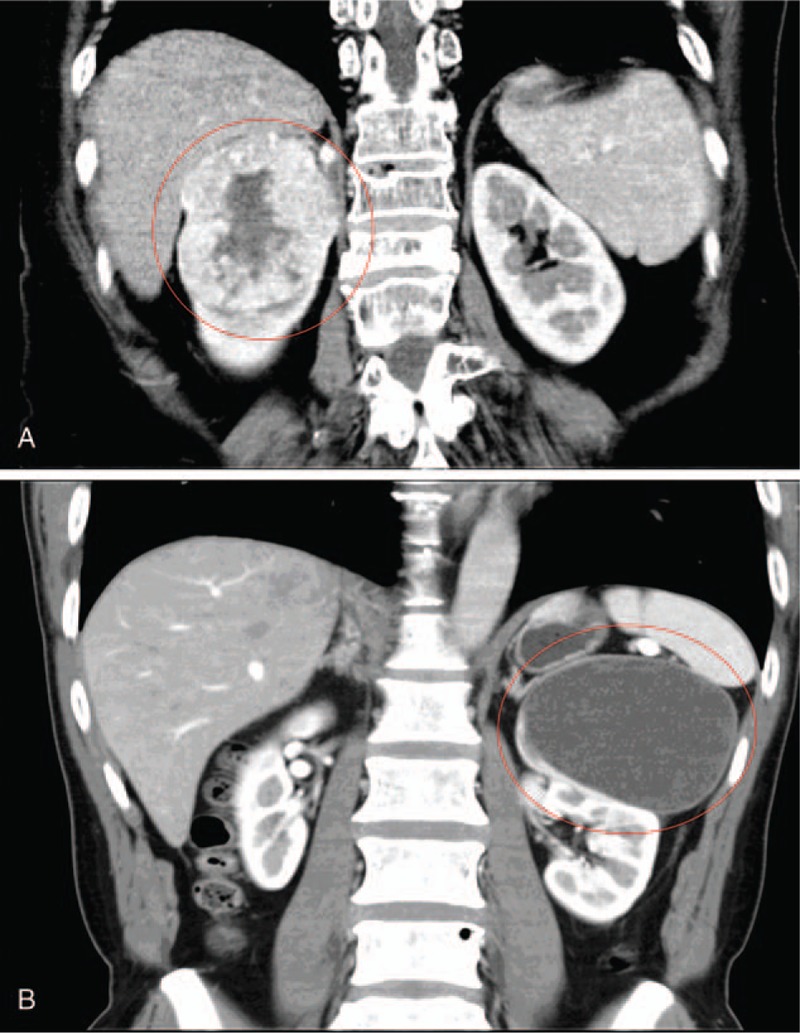
CT image of the patients’ abdominal organs. A, In Patient 1, CT revealed a well-defined solid tissue mass in the right kidney. B, In Patient 2, CT revealed a solid cystic mass in the left kidney. CT  = computed tomography.

The computed tomography (CT) scan revealed a well-defined solid tissue mass in the right kidney that suggested renal cell carcinoma (Fig. 1A). The patient received a radical right nephrectomy without any radiochemotherapy.

**Figure 2:**
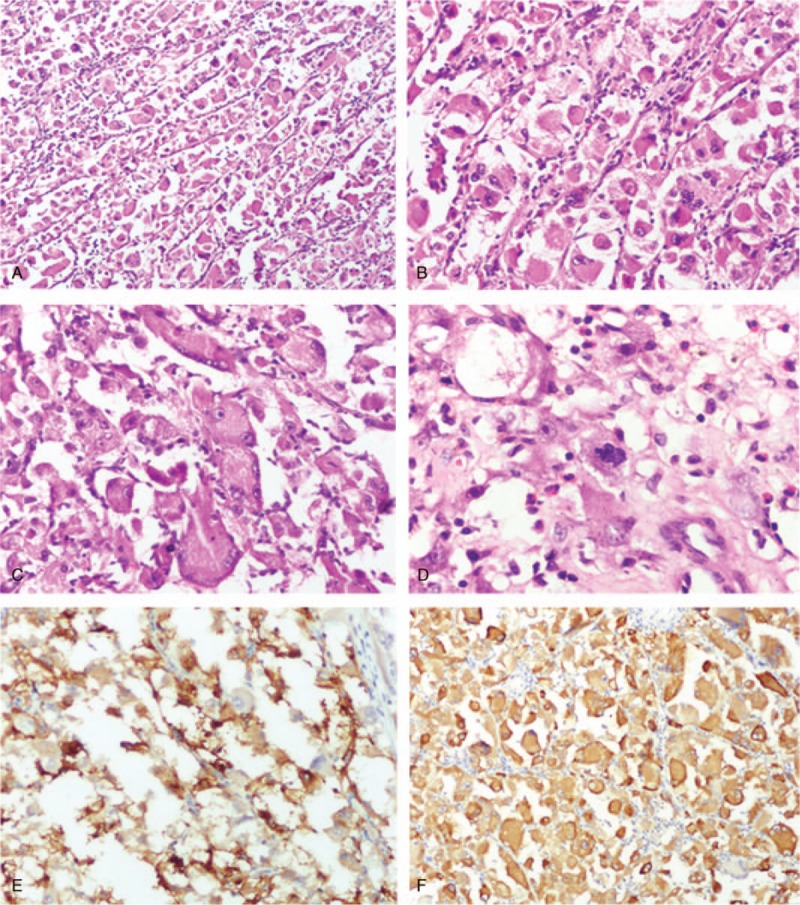
Histochemical and immunohistochemical features of the kidney mass in Patient 1. A–D, Sections of the kidney mass biopsy were made and stained with hematoxylin and eosin. A, Tumor cells were arranged in close nests, separated by glassy collagen fibrils. B, The tumor was characterized by pure epithelioid cells with abundant eosinophilic or granular cytoplasm and prominent nucleoli. C, Multinucleated giant cells. D, Mitotic figure. E and F, Sections of the kidney mass biopsy were assessed with antibodies. E, Tumor cells were focally positive for HMB-45. F, Tumor cells were strongly positive for MelanA. HMB = human melanoma black.

After the nephrectomy, gross examination showed that the kidney parenchyma was partially replaced by a tumor with a volume of 7.5 × 6 × 4 cm^3^. The dissected surface had a solid and soft texture with vague boundary, and a colorful appearance due to necrosis and hemorrhage. Histochemical staining showed that the tumor contained a large portion of necrotic tissue and atypical epithelioid cells with abundant eosinophilic or granular cytoplasm (Fig. 2B). These epithelioid cells were scattered within the tumor, or organized closely in nests separated by glassy collagen fibrils (Fig. 2A). The tumor cells possessed more than 1 round-to-oval atypical nuclei, with irregularly distributed coarse chromatin and prominent nucleoli (Fig. 2C). The mitotic count was about 2 in 50, under high power field (HPF; Fig. 2D). Regretfully, the tumor cells were found infiltrating into the surrounding renal parenchyma.

Immunohistochemical staining showed that the tumor cells tested positive for MelanA (Fig. 2F), were focally positive for HMB-45 (Fig. 2E) and vimentin, and 10% positive for Ki67. Tests for the following were negative: SOX-10, S-100, RCC, CD10, PAX8, PAX2, SMA, desmin, caldesmon, CK, TFE3, CD56, Syn, CgA, P53, and E-cadherin. Based on these findings, malignant EAML was diagnosed.

Thirteen months after the nephrectomy, CT detected a mass in the liver that displayed the same features as the previous tumor in the right kidney. It was speculated that the liver mass was a metastasis from the kidney lesion. No pathological examination was performed for the liver metastasis because the patient refused to provide a biopsy. She also refused to receive any more treatment due to economic stress.

#### 1.1.2 Case 2

A 62-year-old man presented with an untreated left backache of 1 year's duration. The regular physical examination revealed, on ultrasonography, a cystic lesion in the left kidney. A CT scan revealed a cystic solid tumor in the left kidney (Fig. 1B). The kidneys were not palpable under the rib; and no percussion pain or tenderness was detected in the kidney region or ureteral point. He also had no history of TSC. The laboratory examination did not show any abnormality. As requested by the patient, he was treated with a radical left nephrectomy without adjuvant therapy. Ten months after surgery, the patient was living well without any signs of disease.

**Figure 3:**
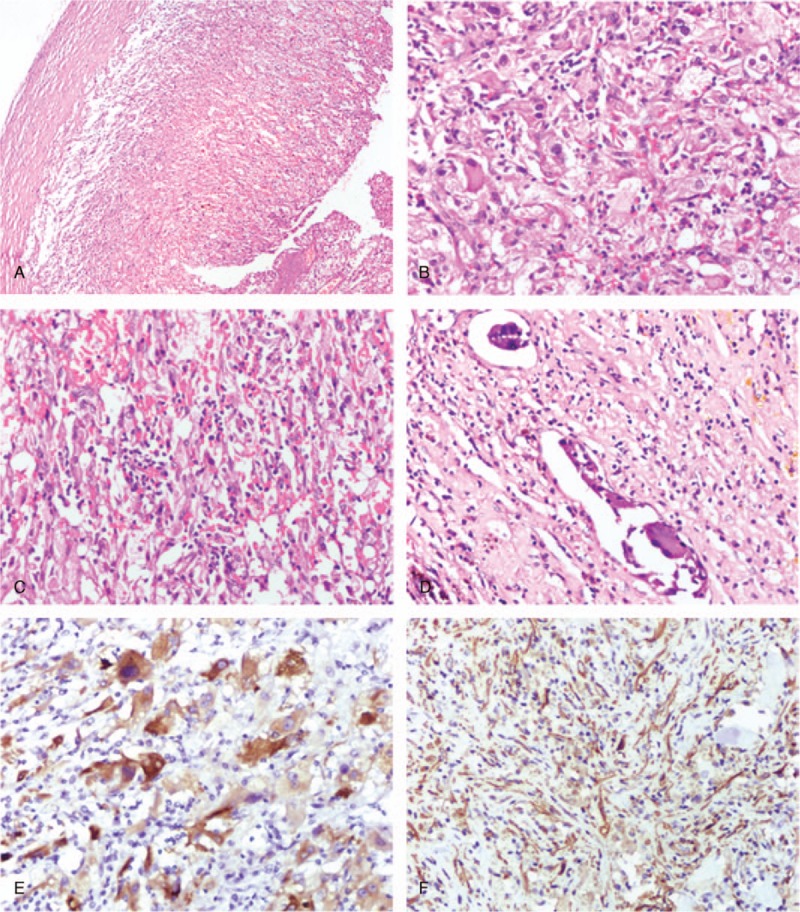
Histochemical and immunohistochemical features of the kidney mass in Patient 2. A–D, Sections of the kidney mass biopsy were made and stained with hematoxylin and eosin. A, The tumor was a solid cystic mass with hemorrhagic necrotic tissue in the cystic cavity. B, The cystic wall was composed predominantly of atypia epithelioid cells with abundant eosinophilic cytoplasm. C, Spindle cells were arranged in bands. D, Lymphovascular invasion. E and F, Sections of the kidney mass biopsy were detected with antibodies. E, Epithelioid cells were focally positive for MelanA. F, Spindle cells were positive for SMA.

Gross examination of the kidney after the nephrectomy showed that the tumor was a well-circumscribed mass with a volume of 7 × 5 × 3.5 cm^3^. The tumor was composed of multiple cysts that contained hemorrhagic necrotic tissue wrapped by a thick cystic membrane. Histology showed cavities of various size in the tumor, surrounded by thick membrane composed predominantly of atypical epithelioid cells with abundant eosinophilic cytoplasm, irregular nuclei, marginal aggregation of chromatin, and prominent nucleoli (Fig. 3A and B). Mitotic figures were counted as 2 per 50 under HPF. In some areas, there were frequent spindle cells arranged in bands, and a large number of slender vessels wrapped by thin membrane were observed in the stroma of the tumor (Fig. 3C). Lymphovascular invasion was also seen in the cystic wall (Fig. 3D).

Immunohistochemical staining confirmed that the epithelioid cells were strongly positive for MelanA (Fig. 3E), and focally positive for HMB-45 and vimentin. Tumor cells were negative for SOX-10, S-100, RCC, CD10, PAX8, PAX2, CK, TFE3, CD56, Syn, CgA, P53, and E-cadherin. Ki67 was positive in about 10% of epithelioid cells. In addition, spindle cells were positive for SMA (Fig. 3F) and focally positive for caldesmon and desmin. Therefore, the final histopathological diagnosis was malignant EAML.

## 2 Discussion

Kidney EAML, mainly composed of epithelioid cells, has the potential to become malignant, with aggressive characteristics. Based on the risk of malignancy, EAML may be classified into 5 groups: none, low, intermediate, high, and malignancy. EAML at low risk of malignancy is ≥7 cm, with ≥50% epithelioid component. EAML at intermediate risk has been associated with TSC, moderate atypia epithelioid cells ≥10%, ≥2/10 HPF, atypical mitosis, and extrarenal extension. High-risk EAML is characterized by severe atypia epithelioid cells ≥10%, a carcinoma-like growth pattern, and tumor necrosis. Malignancy is shown by lymphovascular invasion, lymph node metastasis, or distant metastasis.^[6,8]^

L’Hostis et al^[9]^ in 1999 proposed that only EAML that has spread distantly can be considered malignant. However, some tumors that are not malignant, such as leiomyoma, do spread. Moreover, when metastasis is observed the disease is often at the advanced end stage, and the disease could not be properly treated early. To make an early diagnosis, some researchers have attempted to apply a series of morphological features to indicate progression of malignancy. Brimo et al^[10]^ analyzed 9 cases with local recurrence or distant metastases, and determined that the presence of ≥3 of the following was highly predictive of malignancy: ≥70% atypical epithelioid cells; ≥2 mitotic figures per 10 HPF; atypical mitotic figures; and necrosis. Folpe et al^[2]^ reviewed 26 cases of PEComa, and proposed that any PEComa having ≥2 of the following should be considered malignant: ≥5 cm tumor size; infiltrative; high nuclear grade and cellularity; ≥1 mitotic figure per 50 HPF; necrosis; and vascular invasion.

We reviewed 17 cases of malignant EAML reported with detailed morphological descriptions during the years 2000 to 2017^[4,8,11–23]^ (Tables 1 and 2), and herein propose that 8 features are evidence of malignancy: size ≥ 5 cm; metastasis; infiltration; necrosis; ≥50% atypical epithelioid cells; cytologic atypia; atypical mitosis; and vessel invasion. Of the 17 cases in the literature, 13 with metastasis had ≥5 of these features and were diagnosed as malignant EAML. In addition, 3 cases without metastasis included 7, 6, and 6 malignant features, respectively, and were diagnosed as malignant EAML. Hence, the coexistence of ≥5 of the features noted above may be indicative of malignant EAML. Of note, 1 case without metastasis in our review possessed only 4 malignant features, and thus was not diagnosed as malignant EAML according to our new criteria.

**Table 1:**
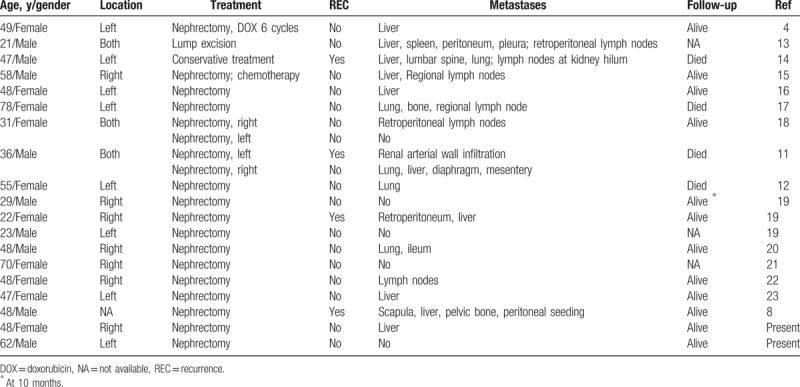
The clinicopathological features of 19 cases of malignant kidney EAML reported during years 2000 to 2017.

**Table 2:**
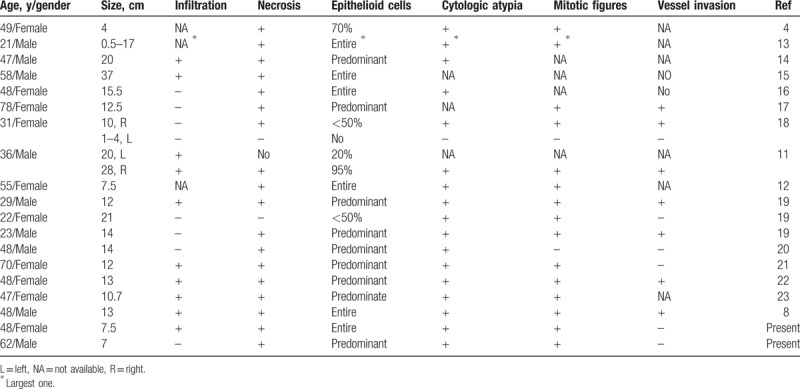
Histopathologic features of malignant kidney EAML reported from year 2000 to year 2017.

The review of 17 cases in the literature led to our proposed criteria for indicators of malignant EAML in kidney, and the 2 cases within our own experience support the validity of these criteria. Specifically, our criteria call for the coexistence of ≥5 of the following 8 features: size ≥ 5 cm; metastasis; infiltration; necrosis; ≥50% atypical epithelioid cells; cytologic atypia; atypical mitosis; and vessel invasion. In our first case (Patient 1), 6 of the 8 features were present: size >7 cm; liver metastasis; infiltration; necrosis; epithelioid atypia; and high mitotic rate. In the second case (Patient 2), there were 5 of the 8 proposed criteria: size >7 cm; necrosis; partial epithelioid atypia; high mitotic rate; and lymphovascular invasion.

Studies in molecular genetics have made some progress in elucidating the genetic mechanism underlying PEComas. It has been suggested that TFE3 (transcription factor binding to IGHM enhancer 3) gene fusions and p53 gene mutation are involved in the genesis of malignant PEComa.^[12,24]^ Moreover, the loss of TSC1 (chromosome 9q34) and TSC2 (chromosome 16p13.3), which are particularly associated with kidney AML,^[25]^ was found to activate the pathway Rheb/mTOR/p70S6K (Ras homolog enriched in brain/ mammalian target of rapamycin/S6 kinase beta-1). It was found that in 19 cases of kidney EAML, 5 were diagnosed as TSC,^[11]^ suggesting that up to 26% of cases were TSC-associated PEComa. Recently, activation of the mTOR pathway was found to be involved in malignant EAML, suggesting that molecules targeting mTOR, such as an mTOR inhibitor, may be used as therapeutic agents to treat malignant EAML.^[26,27]^ However, further studies are needed to assess the curative and side effects of an mTOR inhibitor.

Nephrectomy is still a main treatment strategy for malignant EAML of the kidney. Although it was reported that patients responded well to single-agent doxorubicin,^[4]^ the outcomes have not been validated by long-term and large sample clinical studies, and adjuvant chemotherapy is not often applied for this tumor. Our 2 patients were treated with nephrectomy only, without chemoradiotherapy. Thirteen months after the operation, liver metastasis was found in the first patient. Patient 2 appeared cured at the 10-month follow-up, but it may take more time to determine a prognosis.

In previous reports, malignant EAML progressed to invasion, recurrence, and metastasis, but the prognosis was not always poor. In the 17 cases reviewed in this study, 4 patients died with lung metastases of the tumor; 1 of these with multifocal metastasis. Hence lung metastasis or multifocal metastasis of important organs may suggest a relatively poor prognosis, but this requires closer follow-up of more cases.

In conclusion, 2 cases of kidney malignant EAML are reported here, which support our proposed criteria for indicators of malignant EAML of the kidney. These criteria are the coexistence of ≥5 of the following 8 malignant features: size ≥5 cm; metastasis; infiltration; necrosis; ≥50% atypical epithelioid cells; cytologic atypia; atypical mitosis; and vessel invasion. However, more cases and more studies are required to confirm these judgement criteria. Nephrectomy is currently the common treatment strategy for malignant EAML, but the development of more effective methods is needed, such as chemotherapeutic drugs or targeted therapy strategies.

## Acknowledgments

The authors thank the patients for agreeing to participate in their report and for providing their detailed medical history.

## Author contributions

RZ and Y-QL drafted the manuscript. CZ conducted the histological examinations. C-YC and H-YH performed H&E staining and immunohistochemical staining.

**Methodology:** Chun-Yan Chen, Han-Yu Hu.

**Writing – original draft:** Rui Zhan, Yan-Qing Li.

**Writing – review & editing:** Chun Zhang.
